# Toxicity Assessment of [^177^Lu]Lu−iFAP/iPSMA Nanoparticles Prepared under GMP-Compliant Radiopharmaceutical Processes

**DOI:** 10.3390/nano12234181

**Published:** 2022-11-25

**Authors:** Tania Hernández-Jiménez, Pedro Cruz-Nova, Alejandra Ancira-Cortez, Brenda Gibbens-Bandala, Nancy Lara-Almazán, Blanca Ocampo-García, Clara Santos-Cuevas, Enrique Morales-Avila, Guillermina Ferro-Flores

**Affiliations:** 1 Department of Radioactive Materials, Instituto Nacional de Investigaciones Nucleares, Ocoyoacac 52750, Mexico; 2 Faculty of Chemistry, Universidad Autónoma del Estado de México, Toluca 50180, Mexico; 3 Department of Chemical Analysis, Instituto Nacional de Investigaciones Nucleares, Ocoyoacac 52750, Mexico

**Keywords:** lutetium nanoparticles, [^177^Lu]Lu-nanoparticle toxicity, FAP inhibitor, PSMA inhibitor

## Abstract

The fibroblast activation protein (FAP) is heavily expressed in fibroblasts associated with the tumor microenvironment, while the prostate-specific membrane antigen (PSMA) is expressed in the neovasculature of malignant angiogenic processes. Previously, we reported that [^177^Lu]lutetium sesquioxide-iFAP/iPSMA nanoparticles ([^177^Lu]Lu−iFAP/iPSMA) inhibit HCT116 tumor progression in mice. Understanding the toxicity of [^177^Lu]Lu−iFAP/iPSMA in healthy tissues, as well as at the tissue and cellular level in pathological settings, is essential to demonstrate the nanosystem safety for treating patients. It is equally important to demonstrate that [^177^Lu]Lu−iFAP/iPSMA can be prepared under good manufacturing practices (GMP) with reproducible pharmaceutical-grade quality characteristics. This research aimed to prepare [^177^Lu]Lu−iFAP/iPSMA under GMP-compliant radiopharmaceutical processes and evaluate its toxicity in cell cultures and murine biological systems under pathological environments. [^177^Lu]Lu_2_O_3_ nanoparticles were formulated as radiocolloidal solutions with FAP and PSMA inhibitor ligands (iFAP and iPSMA), sodium citrate, and gelatin, followed by heating at 121 °C (103-kPa pressure) for 15 min. Three consecutive batches were manufactured. The final product was analyzed according to conventional pharmacopeial methods. The Lu content in the formulations was determined by X-ray fluorescence. [^177^Lu]Lu−iFAP/iPSMA performance in cancer cells was evaluated in vitro by immunofluorescence. Histopathological toxicity in healthy and tumor tissues was assessed in HCT116 tumor-bearing mice. Immunohistochemical assays were performed to corroborate FAP and PSMA tumor expression. Acute genotoxicity was evaluated using the micronuclei assay. The results showed that the batches manufactured under GMP conditions were reproducible. Radiocolloidal solutions were sterile and free of bacterial endotoxins, with radionuclidic and radiochemical purity greater than 99%. The lutetium content was 0.10 ± 0.02 mg/mL (0.9 GBq/mg). Significant inhibition of cell proliferation in vitro and in tumors was observed due to the accumulation of nanoparticles in the fibroblasts (FAP+) and neovasculature (PSMA+) of the tumor microenvironment. No histopathological damage was detected in healthy tissues. The data obtained in this research provide new evidence on the selective toxicity to malignant tumors and the absence of histological changes in healthy tissues after intravenous injection of [^177^Lu]Lu−iFAP/iPSMA in mammalian hosts. The easy preparation under GMP conditions and the toxicity features provide the added value needed for [^177^Lu]Lu−iFAP/iPSMA clinical translation.

## 1. Introduction

Tumors are complex pathological arrangements consisting of malignant cells and the tumor microenvironment (stroma). The latter involves the communication of different non-cancerous cells through dynamic networks regulated by chemokines, inflammatory enzymes, growth factors, cytokines, and extracellular matrix components to promote cancer progression [1,2]. The crucial role of specific target proteins associated with the tumor microenvironment is currently receiving extensive attention in molecular imaging and targeted radiotherapy [3,4,5,6]. Since the expression of specific molecular targets in the tumor stroma is dynamic, radiolabeled dual-functional nanoparticles have potential advantages in cancer radiotherapy because of their non-conditioning to the presence of a single molecular target [7,8,9,10,11,12,13].

Fibroblast activation protein (FAP) is heavily expressed in fibroblasts associated with the tumor microenvironment [14]. Likewise, due to its multifunctional protein nature, prostate-specific membrane antigen (PSMA) is expressed in different cancer cells and as part of the tumor microenvironment of several malignant processes, mainly present in the tumor neovasculature because of its involvement in the transduction of signals associated with cell migration and angiogenesis [15,16].

Lutetium-177 is today the most widely used radionuclide in peptide receptor radionuclide therapy due to its availability and nuclear properties useful for theranostic applications (physical half-life of 6.7 d, β-particle emissions with a maximum energy of 497 keV convenient for therapy, and γ-ray emissions of 208 keV, suitable for in vivo imaging) [17,18]. Therefore, many research manuscripts have reported the synthesis of ^177^Lu-labeled metallic and polymeric nanoparticles as radiotherapeutic systems [19,20,21,22,23,24]. However, our group reported the synthesis of [^177^Lu]Lu_2_O_3_ nanoparticles functionalized with peptides for the first time [25,26]. Previously, we found that [^177^Lu]lutetium sesquioxide nanoparticles functionalized with FAP and PSMA inhibitor ligands (iFAP and iPSMA) ([^177^Lu]Lu−iFAP/iPSMA) prevent HCT116 tumor progression in mice [27]. Furthermore, the potential of peptide-coated lutetium-177 nanoparticles (targeting the tumor microenvironment) for treating colorectal liver metastases was demonstrated [27].

The toxicity of nanoparticles depends on their size and shape, surface coating (functionalization with specific molecules), surface area, distribution in biological systems, and their possible aggregation in vivo [28]. In general, the mechanism by which nanoparticles cause toxicity lies in their ability to initiate the formation of genotoxic and cytotoxic reactive species associated with increased inflammation and oxidative stress [29]. Therefore, understanding the toxicity of [^177^Lu]Lu−iFAP/iPSMA nanoparticles in healthy tissues, as well as at the tissue and cellular level in pathological settings, is essential to demonstrate the nanosystem safety for treating patients. In addition, it is equally important to confirm that [^177^Lu]Lu−iFAP/iPSMA can be prepared under good manufacturing practices (GMP) with reproducible pharmaceutical-grade quality characteristics.

This research aimed to prepare [^177^Lu]Lu−iFAP/iPSMA nanoparticles under GMP-compliant radiopharmaceutical processes and evaluate their toxicity in cell cultures and murine biological systems under pathological environments.

## 2. Materials and Methods

### 2.1. Synthesis of Lutetium Nanoparticles

A pilot laboratory scale-up (ISO 6 cleanroom) was performed to obtain batches of 1.0 g of lutetium sesquioxide nanoparticles using the precipitation–calcination method, as previously reported [1]. Briefly, a solution of 2-M NH_4_OH/(NH_4_)_2_CO_3_ 1:1 (*v/v*) was added dropwise to 5 mL of an aqueous solution (type-I water) of LuCl_3_ (0.7 M) (99.99% trace metals basis; anhydrous powder; Sigma-Aldrich: Saint Louis, MO, USA) until the mixture reached a pH of 9, with a milky appearance (white precipitate). Subsequently, it was stirred for one hour. Then, the mixture was centrifuged in 50-mL conical tubes, and the solid residue was washed five times by centrifugation (2500× *g*/10 min) with type-I water (volumes of 15 mL). Next, the residue obtained was dried at 60 °C for 24 h. After drying, the solid was mechanically pulverized using a porcelain mortar and pestle until a fine powder was obtained. Finally, the powder was calcined at 1100 °C for 24 h and mechanically crushed using a porcelain mortar and pestle until a fine and homogeneous power was achieved (apparent density of 2.1 g/mL). The final product was analyzed by infrared spectroscopy (Fourier transform infrared: ATR platform; Mod. 2000; PerkinElmer: Waltham, MA, USA) and transmission electron microscopy (JEOL TEM; Mod. JEM 2010-HT: Peabody, MA, USA). In addition, a nanoparticle size analyzer (Nanotrac; Mod. Wave I: Montgomeryville, PA, USA) was used to evaluate the hydrodynamic nanoparticle size by dynamic light scattering. The general pharmacopeial analysis method, gas chromatography (GC System; Mod. 7890B; Agilent Technologies: Santa Clara, CA, USA), was performed on the Lu_2_O_3_ nanoparticles to assess the content of residual solvents.

### 2.2. Preparation and Quality Control of [^177^Lu]Lu−iFAP/iPSMA

Three consecutive batches of [^177^Lu]Lu−iFAP/iPSMA were manufactured. First, Lu_2_O_3_ nanoparticles (12 mg) were activated by neutron irradiation (flux of $3\times 10^{13}$ n/s^.^cm^2^; Triga Mark III nuclear reactor) for 60 h (2 cycles of 30 h with an interval of 5 d between them) at the National Institute for Nuclear Research, Mexico.

**Figure A1:**
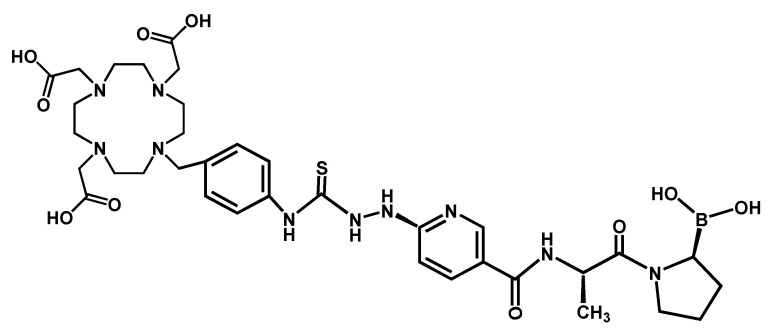
Chemical structure of 2,2′,2″,2‴-(2-(4-(2-(5-(((S)-1-((S)-2-boronopyrrolidin-1-yl)-1-oxopropan-2-yl)carbamoyl)pyridin-2-yl)hydrazine-1-carbothioamido)benzyl)-1,4,7,10-tetraazacyclododecane-1,4,7,10-tetrayl)tetraacetic acid (DOTA−iFAP: iFAP) [30].

**Figure A2:**
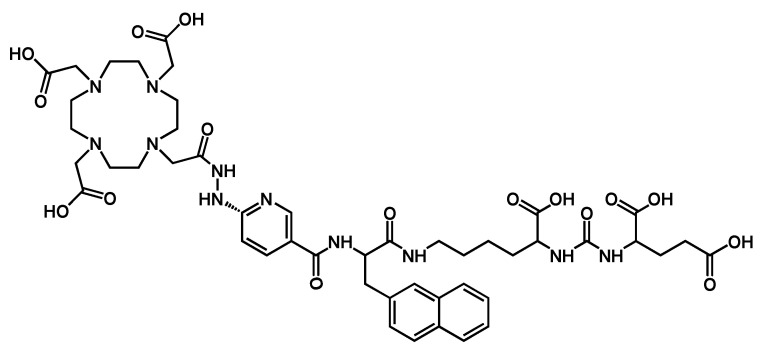
Chemical structure ((1-carboxy-5-(3-(naphthalen-2-yl)-2-(6-(2-(2-(4,7,10-tris (carboxymethyl)-1,4,7,10-tetraazacyclododecan-1-)acetyl)hydrazinyl)nicotinamido)propanamido)pentyl)carbamoyl)glutamic acid (DOTA−iPSMA: iPSMA) [31].

After 24 h of irradiation, [^177^Lu]Lu_2_O_3_ ($3.5\times 10^{15}$ nanoparticles; average size of 23.4 nm; 0.9 GBq/mg) was suspended in 20 mL of a sterile sodium citrate solution (5 mg/mL; 25-mL vial). Next, 50 µL (1 mM; $2.8\times 10^{16}$ molecules) of 2,2′,2″,2‴-(2-(4-(2-(5-(((S)-1-((S)-2-boronopyrrolidin-1-yl)-1-oxopropan-2-yl)carbamoyl)pyridin-2-yl)hydrazine-1-carbothioamido)benzyl)-1,4,7,10-tetraazacyclododecane-1,4,7,10-tetrayl)tetraacetic acid (DOTA-iFAP: FAP inhibitory ligand) (Appendix A) (Figure A1) [30], and 50 µL (1 mM; $2.8\times 10^{16}$ molecules) of ((1-carboxy-5-(3-(naphthalen-2-yl)-2-(6-(2-(2-(4,7,10-tris(carboxymethyl)-1,4,7,10-tetraazacyclododecan-1-l)acetyl)hydrazinyl)nicotinamido)propanamido)pentyl)carbamoyl)glutamic acid (DOTA-iPSMA: PSMA inhibitory ligand conjugated) (Appendix A) (Figure A2) [31] were added. After shaking for 2 min, 1 mL of gelatin (18 mg/mL) was incorporated. The vial was sealed with an aluminum cap, followed by sterilization at 121 °C (103-kPa pressure) for 15 min. Later, the sealed vial was shaken in a vortex for 5 min, followed by 5 min in an ultrasonic bath; this alternating shaking process was repeated three times. Next, the radiocolloidal solution of [^177^Lu]Lu−iFAP/iPSMA was dispensed in three aliquots of 7 mL each (using sterile 10-mL syringes), into apyrogenic and sterile 23-mL glass vials (sealed with aluminum caps) containing 13 mL of injectable-grade 0.9 % NaCl. The vials were stirred, and the activity (3.4 ± 0.2 GBq/vial) was measured in an ionization chamber (Capintec dose calibrator; Mod. CRC-55tR: Florham Park, NJ, USA). The processes were carried out in areas with GMP certification. In particular, most of the operations were carried out in a shielded cell (Comecer; Mod. Musa: Castel Bolognese, RA, Italy) equipped with HEPA terminal filters (99.997% efficiency; laminar flow system) and a UV lamp, and programmed with a vertical laminar flow (0.3 m/s; ISO Class-5 degree of cleanliness).

The final radiocolloidal formulations were subjected to sterility and bacterial endotoxin tests in agreement with general pharmacopeial methods [32]. Radionuclide and radiochemical purity were verified by gamma spectroscopy (Atomtex; Mod. AT1315 gamma beta spectrometer: Minsk, Republic of Belarus) and ultracentrifugation (2500× *g* for 20 min; 100,000 MW cut-off; Amicon Ultra; Millipore: Burlington, MA, USA), respectively. After centrifugation, [^177^Lu]Lu−iFAP/iPSMA nanoparticles remained in the filter, while [^177^Lu]-lutetium oxide (not in nanoparticle form) and [^177^Lu]-lutetium chloride passed through the filter. After the complete decay of lutetium-177, one sample was used to verify the nanoparticle size distribution by DLS and TEM, Z potential was determined by DLS, nanoparticle functionalization via IR spectroscopy, and heavy metal content by ICP-MS (Quadrupole mass spectrometer; Mod. 7700×; Agilent Technologies: Santa Clara, CA, USA) employing certified reference materials (CRM) (high-purity standards: North Charleston, SC, USA). ICP-MS results were correlated to the final radiocolloidal activity and expressed as µg/GBq.

To quantify lutetium in the Lu−iFAP/iPSMA colloidal solution, a total reflection X-ray fluorescence (TXRF) analysis was performed (Bruker spectrometer, Mod. S2 PICOFOX XRF: Billerica, MA, USA). Rubidium (1000 mg/L) and lutetium (1000 mg/L) (CRM) were used as internal standards. The nanoparticle samples were shaken in a vortex for 2 min. To an aliquot of 0.9 mL of each sample, 0.1 mL of Rb (CRM) was added and stirred for 2 min. Next, 10 µL was deposited in a quartz reflector and dried on a plate by heating at 60 °C. Each sample was prepared and analyzed in triplicate. A quality control solution with reagent blanks was prepared to evaluate the accuracy of the method: 9 mL of sodium citrate (0.5 mg/mL) + 100 μL of gelatin (18 mg/mL) + 0.9 mL of Lu (CRM) + 0.1 mL of Rb (CRM). The solution was shaken for 1 min, then 10 µL was spotted on a quartz reflector and dried on a hot plate at 60 °C. The quality control solution was prepared and analyzed in triplicate. The internal standard addition method obtained the elemental concentration using the SPECTRA 7^®^ software (Version 7.5.3.0).

### 2.3. Cell Culture

The human colorectal cancer cell line HCT116 (ATCC^®^ CCL-247™) and U-937 macrophages (human immune cells) (ATCC^®^ CRL-1593.2™) were cultured in RPMI-1640 (ATCC^®^ 30-2001™) medium (ATCC, Manassas, VA, USA). Cells were grown under an atmosphere of CO_2_ (5%) at 37 °C. The medium was supplemented with 1% penicillin/streptomycin solution and 10% fetal bovine serum.

### 2.4. Cell Viability

Considering the relevant role of macrophages in nanoparticle metabolism [28,29], the effect on viability of U-937 macrophages [33] and HCT116 cancer cells exposed to [^177^Lu]Lu−iFAP/iPSMA was assessed. In brief, a set (Roche: Basel, Switzerland) of XTT (2,3-bis [2-methoxy-4-nitro-5-sulfophenyl]-2H-tetrazolium-5-carboxyanilide) reagents was used following the manufacturer’s indications. U937 macrophages or HCT116 colorectal cancer cell lines (10^4^ cells/well) were seeded in 96-well microtiter plates. After treatment with [^177^Lu]Lu−iFAP/iPSMA nanoparticles (60 μL; 5 MBq; 0.01 mg of nanoparticles), the viability was measured at 24, 48, and 72 h (incubation at 85% humidity, in 5% CO_2_, and 37 °C). The absorbance in each well was recorded on a microplate reader (Epoch; BioTek Instruments; Winooski, VT, USA) at 450 nm. The untreated cells were used as the control group (100% viability).

### 2.5. Coculture of Cancer Cells and Macrophages

HCT116 colorectal cancer cells were cultured with U-937 macrophages in RPMI-1640 medium under an atmosphere of CO_2_ (5%) at 37 °C. The medium was supplemented with 1% penicillin/streptomycin solution and 10% fetal bovine serum.

[^177^Lu]Lu−iFAP/iPSMA uptake kinetics in cocultures of U937 macrophages and HCT116 cancer cells was analyzed by fluorescence microscopy. After incubation with the [^177^Lu]Lu−iFAP/iPSMA nanoparticles (60 μL; 5 MBq; 0.01 mg of nanoparticles) for 1, 3, and 24 h, the cells were fixed with 4% paraformaldehyde for 20 min, permeabilized with 0.5% Triton X-100, and 1% bovine serum albumin was used for cell blocking. DAPI was used for nuclei staining and fluorescence microscopy (MeijiTechno; Mod. MT6200: Saitama, Japan) for intracellular fluorescence intensity observation.

### 2.6. Administration of [^177^Lu]Lu−iFAP/iPSMA in HCT116 Tumor-Bearing Mice

Athymic mice (weight of 21 ± 1 g; 8 weeks old; nude female mice) (n = 10) were subcutaneously injected into the right flanks with HCT116 cells ($1\times 10^{6}$ cells suspended in 0.2 mL PBS) for tumor induction. Studies were performed under ethical regulations for handling of laboratory animals (Official Mexican Norm NOM-062-ZOO-1999).

Five nude mice with visibly developed tumors (0.50 ± 0.2 g) were intravenously injected (tail vein) with [^177^Lu]Lu−iFAP/iPSMA nanoparticles (100 μL; 15 MBq; 0.017 mg of nanoparticles; ~0.85 mg of nanoparticles/kg of mouse weight) at day 1, 4, 7, 10, and 14 for a total of five doses. In addition, a group of five untreated tumor-bearing mice was used as a negative control. Tumor growth was monitored twice weekly using the length (L) and width (a) measurements in Equation (1):

(1) V=π6×(L)×(a2)

After 28 d (two weeks after the last dose), mice were sacrificed to draw blood samples for quantitation of creatinine and liver and heart enzymes. Tumors and the liver, spleen, kidney, lung, brain, and ovary were dissected and prepared for histopathological or immunohistochemistry studies.

### 2.7. Histopathological Evaluation

Tumors and the liver, spleen, kidney, lung, brain, and ovary from mice were fixed with 4% formaldehyde for 24 h at room temperature, embedded in paraffin, and sectioned in 4–5 μm-thick sections using a microtome. Paraffin sections of tumor samples were deparaffinized in xylene and rehydrated in a series of graded alcohols. Dewaxed tissue sections were stained with hematoxylin and eosin (H&E). Sections were evaluated using a light microscope (magnification of 400× and 1000×) (Zeiss; Mod. Axioscope: Oberkochen, Germany). Images were acquired with a digital camera (5MP high-speed color; AmScope; Mod. Mu500: Irvine, CA, USA).

### 2.8. Immunohistochemistry

#### 2.8.1. Ki67 and pEGFR Expression

Paraffin sections of tumor samples were deparaffinized in xylene and rehydrated in a series of graded alcohols. Antigens were retrieved in 0.1 M EDTA-Tris buffer. Samples were incubated in 0.9% H2O2 for 5 min, followed by a one-hour blocking period in 1% BSA in PBS. Slides were incubated for one hour at room temperature with anti-Ki67 (1:100; Biocare Medical: Concord, CA, USA) and anti-pEGFR (1:100; phosphorylated Tyr1068 epidermal growth factor receptor; Biocare Medical: Concord, CA, USA), washed, and incubated in MACH1 Universal HRP-polymer (Biocare Medical: Concord, CA, USA) for one hour at room temperature. Then, samples were developed with Betazoid DAB Chromogen Kit (Biocare Medical: Concord, CA, USA), counter-stained with hematoxylin, and mounted with synthetic resin solution. Sections were evaluated using an optical microscope (magnification of 400× and 1000×) (Zeiss, Mod. Axioscope: Oberkochen, Germany). Images were acquired with a digital camera (5MP high-speed color; AmScope Mod. Mu500: Irvine, CA, USA).

#### 2.8.2. PSMA and FAP Expression

Dewaxed and hydrated tumor sections (stained with H&E to identify representative areas) were incubated in 0.9% H_2_O_2_ for 5 min, followed by one hour of blocking in 1% BSA in PBS. Immunohistochemical staining was performed with the mouse anti-PSMA monoclonal antibody (1:250 dilution; GCP-04 GeneTex: Irvine, CA, USA) or the rabbit anti-FAP polyclonal antibody (1:100 dilution; PA5-99458 Invitrogen: Waltham, MA, USA). Random fields for tumor sections were evaluated using a light microscope (magnification of 400× and 1000×) (Zeiss, Mod. Axioscope: Oberkochen, Germany). Images were acquired with a digital camera (5MP high-speed color; AmScope Mod. Mu500: Irvine, CA, USA).

### 2.9. Creatinine and Enzyme Quantitation

The blood samples of mice, obtained by cardiac puncture, were used to evaluate the kidney function through creatinine quantitation (picrate method), the liver function through alanine aminotransferase (ALT) and aspartate aminotransferase (AST) quantitation, cytotoxic tissue damage (LDH, lactate dehydrogenase), and cardiac function (CK). The enzymes were quantified using conventional in vitro diagnostic assays (Roche/Hitachi Cobas 8000 modular analyzer: Basel, Switzerland).

### 2.10. Genotoxicity Assay

The genotoxic effect of [^177^Lu]Lu−iFAP/iPSMA was performed through the identification of micronuclei (damaged chromosomes not incorporated into the nucleus after cell division) in immature erythrocytes from bone marrow in healthy BALB/c mice (weight of 23 ± 1 g; 10 weeks old; male mice) treated with nanoparticles administered intravenously (100 μL; 15 MBq; 0.017 mg of nanoparticles; ~0.85 mg of nanoparticles/kg of mouse weight) (n = 12). Untreated mice were used as a negative control (n = 12). In addition, colchicine was administered at 1 mg/kg as a positive control (n = 12) for micronucleus formation. In groups of four, mice were sacrificed at 24, 48, and 168 h after each treatment to obtain bone marrow cells, which were stained with Giemsa-Wright (Diff-Quick; Harleco; Gibbstown, NJ, USA). Two thousand polychromatic erythrocytes (PCE) per mouse were counted using a light microscope (Zeiss, Mod. Axioscope: Oberkochen, Germany) at a magnification of 1000× to determine the frequency of polychromatic erythrocytes with micronuclei.

### 2.11. Statistical Analysis

Differences between groups in the cytotoxicity, genotoxicity, and evaluation of HCT116 tumor growth assays were analyzed using the Student’s t-test (*p* < 0.05 was established as significant).

## 3. Results

**Figure 1:**
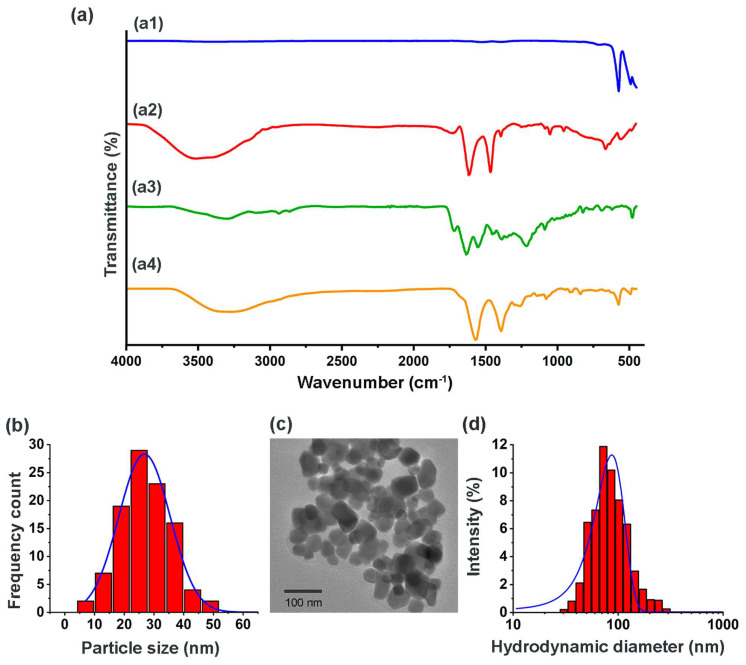
Chemical characterization of [^177^Lu]Lu−iFAP/iPSMA nanoparticles formulated under GMP conditions (evaluation after the complete decay of ^177^Lu). (**a**) Infrared spectra: (a1) Lu_2_O_3_ nanoparticles, (a2) iFAP, (a3) iPSMA, and (a4) Lu−iFAP/iPSMA; (**b**) size distribution of Lu−iFAP/iPSMA by TEM; (**c**) TEM micrograph of Lu−iFAP/iPSMA; (**d**) size distribution of Lu−iFAP/iPSMA by DLS (hydrodynamic diameter: particle diffusion within the fluid).

FT-IR, DLS and TEM spectra of lutetium nanoparticles analyzed before and after being irradiated and prepared under GMP conditions, indicated that the morphology, size, and chemical composition of the Lu−iFAP/iPSMA nanosystem remained stable as a pharmaceutical formulation. Figure 1 shows the chemical characteristics of the formulated nanoparticles. Figure 1a compares the IR spectrum of Lu−iFAP/iPSMA nanoparticles with regard to the employed raw materials: (1) infrared spectrum of lutetium oxide nanoparticles (Figure 1a1), where a narrow and intense band at 574 cm^−1^ of the Lu-O vibration was indicative of the nanometric size, the crystalline purity and the adequate crystallization of the nanomaterial [25,26]; and (2) infrared spectra of the targeting ligands iFAP (Figure 1a2) (Figure A1) and iPSMA (Figure 1a3) (Figure A2).

In the iFAP spectrum (Figure 1a2), the pyridine ring’s ʋ(-NH) vibration was observed at 3364 cm^−1^. The ʋs(-OH) vibration from boronic acid was detected at 3252 cm^−1^, as well as (-NH) from hydrazine (3500 cm^−1^). Another band from boronic acid associated with the B-O vibration was found at 1442 cm^−1^. The asymmetric and symmetric vibrations of the -CH(CH_3_) group were assigned at 2892 cm^−1^ and 2828 cm^−1^, respectively. The –(C=O) vibrational band, present in the carboxylic acids of DOTA and the tertiary amide, was observed at 1651 cm^−1^. The aromatic ring’s (C-H) stretching vibration was detected at 3034 cm^−1^. The in-plane and out-of-plane vibrations for the same group were assigned in the region from 1206 cm^−1^ to 1142 cm^−1^ and from 923 cm^−1^ to 634 cm^−1^, respectively. Finally, the C-N and C-N-H groups of amides II presented a vibrational band between 1412 and 1337 cm^−1^. Regarding the iPSMA ligand spectrum (Figure 1a3), a broad band at 3284 cm^−1^ was assigned to ʋs(-NH) and ʋs(-OH). The asymmetric and symmetric vibrations of (-CH_2_) were observed at 2987 cm^−1^ and 2923 cm^−1^, respectively. At 2537 cm^−1^ and 1652 cm^−1^, the ʋ(-OH) and ʋ(-C=O) vibrations of -COOH of DOTA were found. The band observed at 1551 cm^−1^ was correlated with the ʋ(-CN) of urea from iPSMA and to the ʋ(-NH) vibration of amides II. Between 1442 cm^−1^ and 1347 cm^−1^, the vibrations of the -NH, C=CC, and C-N groups were found. In addition, the C-N-C vibrations of the DOTA structure occurred at 1257 cm^−1^ and 1149 cm^−1^.

The infrared spectrum of the Lu−iFAP/iPSMA nanoparticles (Figure 1a4) evidenced the changes in the chemical environment due to the interaction among the ligand molecules and the nanoparticles’ surface. First, at 3284 cm^−1^, a band assigned to the ʋs(-NH) and ʋs(-OH) vibrations of iPSMA, as well as to the ʋ(-NH) vibration of the iFAP pyridine ring, was observed. The said band is broader with regard to the bands present in the free iFAP and iPSMA ligands. In the region from 2422 cm^−1^ to 2148 cm^−1^, an overlap of bands of the assigned groups for both ligands was observed. The asymmetric and symmetric vibrations of the aliphatic chains of both molecules presented bands at 2978 cm^−1^ and 2923 cm^−1^. The band located at 1389 cm^−1^ corroborated the presence of iFAP on the surface of the nanoparticles, as it was assigned to the B-O vibration from boronic acid. The presence of iPSMA was confirmed with the band at 1570 cm^−1^, associated with the ʋ(-CN)-urea group.

**Figure A3:**
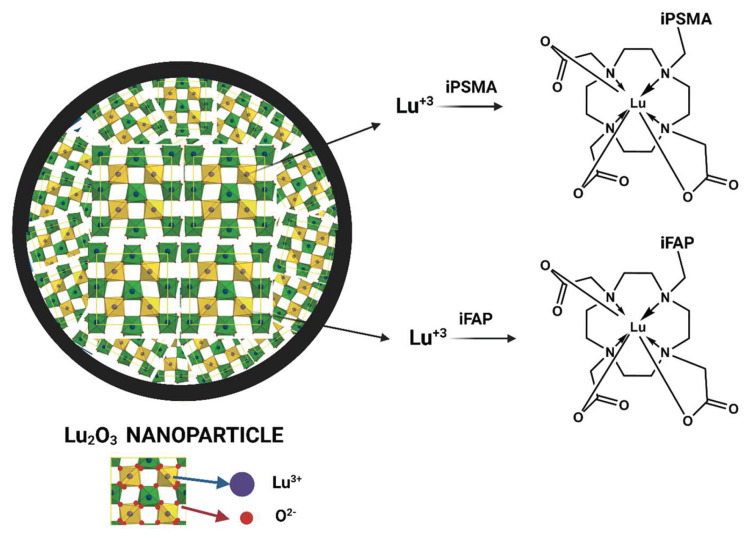
Schematic structure of the Lu−DOTA−iPSMA/iFAP complexes produced on the surface of lutetium sesquioxide nanoparticles.

Furthermore, nanoparticle functionalization with both inhibitor ligands was demonstrated with the shifts to lower energy (~80 cm^−1^) of ʋ(-COOH) from DOTA due to their interaction with the Lu^3+^cation (Figure A3). Likewise, the Lu-N interaction was found at 1257 cm^−1^, which was shifted with regard to the band at ~1321 cm^−1^ of the free tertiary amines in the DOTA moiety of iFAP and iPSMA. Both displacements in energy were evidence of the coordinated covalent bond formation between the Lu^3+^ cations on the surface of the nanoparticles and the DOTA chelator of iFAP and iPSMA (Figure A3). Finally, the Lu-O stretching vibrational band, associated with the internal structure of the nanoparticles, was observed at 574 cm^−1^. The Lu-O vibrational band also confirms that the formation of the final coordination complex was carried out by the chemical interaction between Lu^3+^ and the electron pairs of the nitrogens (-NH) and oxygens (-OH) of DOTA [25,26,27,30].

TEM micrographs (Figure 1c) showed a spherical morphology with uniform and homogeneous distribution of the nanoparticles. The average diameter obtained from measurements of ~600 particles with the ImageJ program was 23.4 ± 7.9 nm with a monomodal and monodisperse distribution (Figure 1b); these results corroborated the formation and stability of the lutetium nanoparticles, in accordance with our previous reports [25,26]. With the average nanoparticle size value, and using the parameters reported for the crystalline cell of the nanomaterial (body-centered cubic space group la3, a ≈ 10.36 Å and a z = 16), the number of binding sites (Lu^3+^) on the surface of the Lu_2_O_3_ nanoparticles was calculated. It was considered that the cubic unit cell is made up of 80 ions, 48 of which correspond to the O_2_^−^ anion, while the remaining 32 represent the Lu^3+^ cations located in C2 and C3i possible sites (~8 cations of Lu^3+^ available per face, considering that those located in the cubic edges and vertices are available in two or three faces) [34,35,36,37].

The volume of each cubic unit cell was calculated as 112.48 nm^3^ (a^3^). The average size of one Lu_2_O_3_ nanoparticle (23.4 nm^2^ of diameter) represents a volume of 6709 nm^3^, which indicated that approximately 60-unit cells ($1.33\times 10^{-22}$ molecules of Lu_2_O_3/_cell) form one Lu_2_O_3_ nanoparticle. Likewise, the surface area of one nanoparticle was calculated as 1720 nm^2^, where 159 faces (a^2^ = 10.8 nm^2^/face) of cubic unit cells could be located as available sites to bind iFAP and iPSMA ligands through their DOTA moiety. Using the molecular weight of Lu_2_O_3_ and Avogadro’s number, the estimated value of the mass of one Lu_2_O_3_ nanoparticle was $5.3\times 10^{-20}$ g. Thus, the total number of nanoparticles and Lu^3+^ cations available on the surface of lutetium sesquioxide nanoparticles (12 mg) were calculated, which corresponded to $3.5\times 10^{15}$ Lu_2_O_3_ nanoparticles and $4.5\times 10^{18}$ Lu^3+^ cations on the surfaces (1272 cations of Lu^3+^/nanoparticle surface). These values indicated that each nanoparticle could easily be functionalized on its surface with eight iFAP molecules ($2.8\times 10^{16}$ molecules added to the formulation containing 12 mg of Lu_2_O_3_ nanoparticles) and eight iPSMA molecules ($2.8\times 10^{16}$ molecules added to the formulation). Therefore, no steric hindrance occurred due to the molecular size of iFAP and iPSMA (from 2 to 3 nm). In addition, the DOTA chelator of both molecules has the same probability of complexing with the available Lu^3+^ cations.

On the other hand, the hydrodynamic diameter obtained by DLS was 95 ± 22 nm, with a monomodal and monodisperse distribution (Figure 1d) and a Zeta potential value of −29 mV, which also indicated adequate colloidal stability.

The quality control results of three batches indicated that [^177^Lu]Lu−iFAP/iPSMA nanoparticles were produced with appropriate pharmaceutical characteristics in a reproducible manner (Table 1).

**Figure 2:**
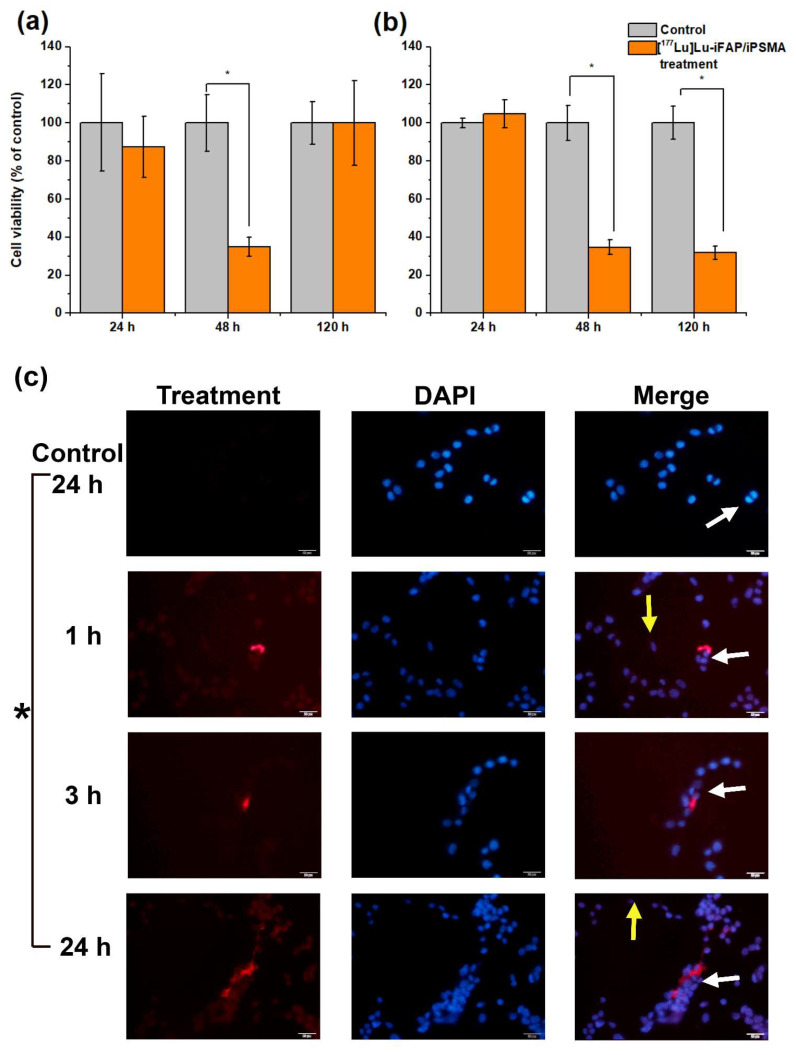
Cell viability of (**a**) U-937 macrophages and (**b**) HCT116 colorectal cancer cells exposed to [^177^Lu]Lu−iFAP/iPSMA nanoparticles for 24, 48, and 120 h (orange bar). Untreated cells were considered the control group (gray bar). (**c**) Representative fluorescence micrographs (400×) of a coculture of colorectal cancer cells and macrophage immune cells. The coculture was treated with [^177^Lu]Lu−iFAP/iPSMA nanoparticles for 1, 3, and 24 h. Control: cells without treatment at 24 h (red optical density of 10.95 ± 2.23). **Red**: emission of nanoparticles with an optical density of 29.57 ± 5.35, 34.17 ± 1.76, 79.85 ± 1.07 at 1, 3, and 24 h, respectively; **Blue**: DAPI (cell nuclei) and merged images. Yellow arrows point to colorectal cancer cells (tendency toward an oval shape). White arrows indicate macrophages (more prominent and more rounded than cancer cells). Of note is the significant presence of nanoparticle clusters associated with the cytoplasm of macrophages, whose accumulation increases markedly over time in parallel with macrophage proliferation. * Statistically significant difference (*p* < 0.05).

Kupfer cells and splenic macrophages are the cellular entities that mainly accumulate nanoparticles in both healthy tissues and tumors [28,29]. That is why the cytotoxicity of [^177^Lu]Lu−iFAP/iPSMA was evaluated in a macrophage model (U-937 human immune cells). Due to the high radio-resistance of macrophages [38,39], the results revealed that their viability was not irreversibly affected by radionanoparticle treatment (Figure 2a), as compared to HCT116 colorectal cancer cell behavior (Figure 2b). Furthermore, the kinetics of [^177^Lu]Lu−iFAP/iPSMA uptake by U-937 and colorectal cancer cells in a coculture also demonstrated that macrophages are those that mostly take up nanoparticles over time, and that their viability was not only not affected by lytic or apoptotic cell processes induced by radiation or nanoparticles, but their proliferation was also increased (Figure 2c).

**Figure 3:**
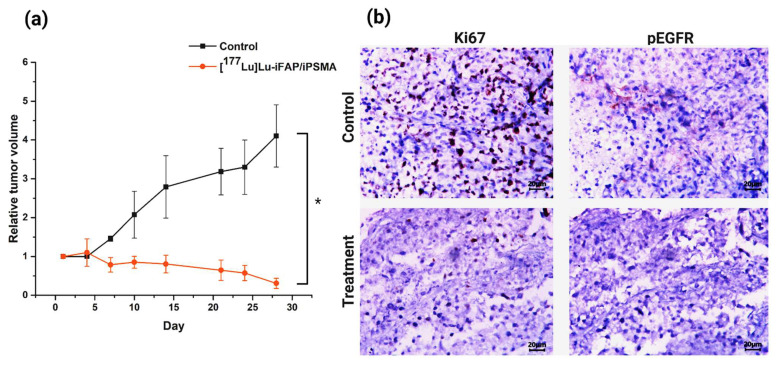
(**a**) Effect of [^177^Lu]Lu−iFAP/iPSMA treatment on HCT116 colorectal cancer xenograft tumors in nu/nu mice. Note a significant decrease in the relative tumor volume in mice treated with the nanoparticles compared to the control group. (**b**) Representative light micrographs (magnification of 400×) of immunohistochemical staining (dark brown color) for cell proliferation markers Ki67 and pEGFR in colorectal cancer xenograft tumors. Note the significant effect of [^177^Lu]Lu−iFAP/iPSMA on the decrease of tumor cell proliferation with regard to the control group. * Statistically significant difference (*p* < 0.05).

The toxicity of [^177^Lu]Lu−iFAP/iPSMA nanoparticles was evaluated in vivo in nu/nu mice bearing subcutaneous colorectal cancer xenograft tumors. The tumor growth rate in mice treated with [^177^Lu]Lu−iFAP/iPSMA was significantly lower than in the control group (*p* < 0.05) (Figure 3a). In addition, tumor progression was evaluated using cell proliferation (Ki67) and malignancy (pEGFR) biomarkers. Immunohistochemical analysis of the expression of both markers showed that the treatment with [^177^Lu]Lu−iFAP/iPSMA reduces the proliferation of colorectal tumor cells (Figure 3b). In contrast, both biomarkers were significantly raised in control tumors (Figure 3b).

**Figure 4:**
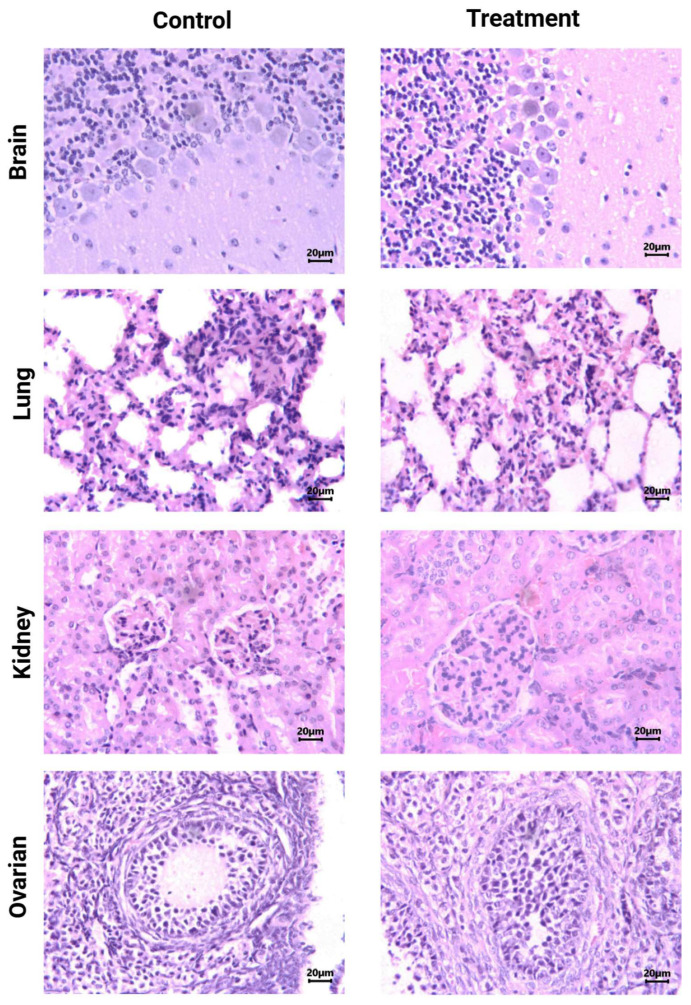
Histological analysis (H&E staining). Light micrographs of representative sections at 400× magnification of brain, lung, kidney, and ovarian tissues from [^177^Lu]Lu−iFAP/iPSMA-treated and untreated (control) mice. Note that tissue toxicity was not found in neither treated nor control mice.

**Figure 5:**
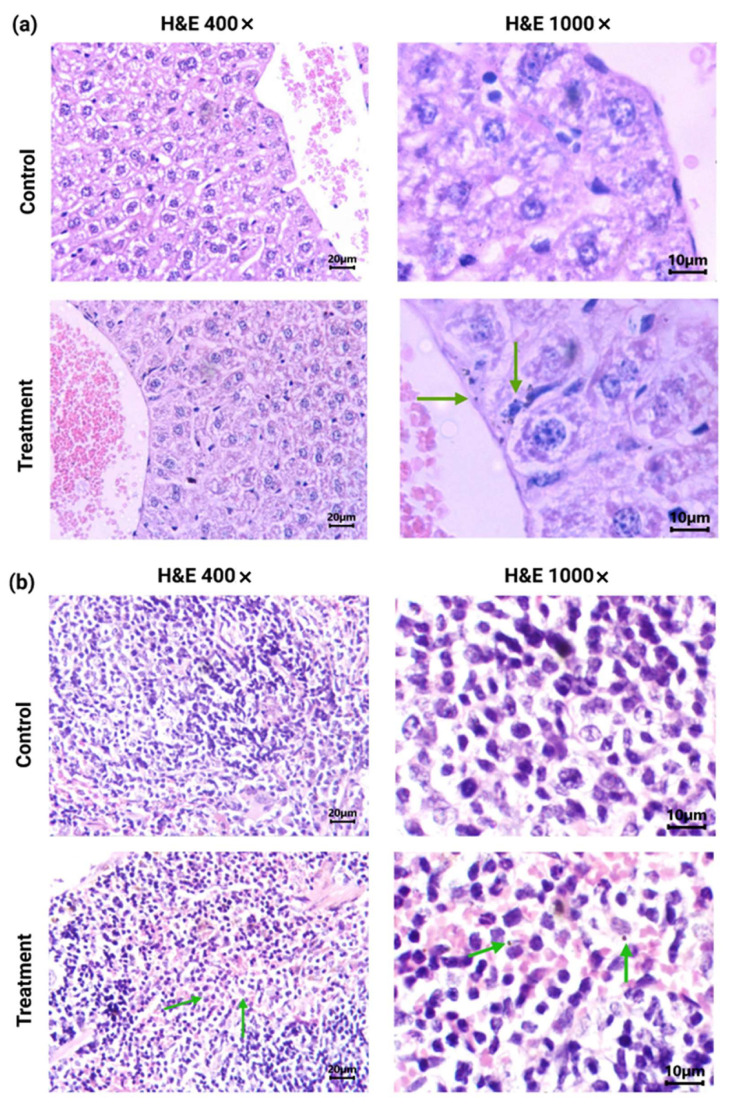
Histological analysis (H&E staining) (light micrographs): (**a**) representative sections at 400× and 1000× magnification of the liver from [^177^Lu]Lu−iFAP/iPSMA-treated and control mice; (**b**) representative sections at 400× and 1000× magnification of the spleen from [^177^Lu]Lu−iFAP/iPSMA-treated and control mice. Note the presence of nanoparticle clusters, especially within macrophages (green arrows).

Histological analysis (H&E staining) of brain, lung, kidney, and ovarian tissues from [^177^Lu]Lu−iFAP/iPSMA-treated and control mice evidenced no tissue toxicity (Figure 4). As previously reported [27], [^177^Lu]Lu-iFAP and [^177^Lu]Lu-iPSMA biokinetic profiles showed that the liver is the organ with the highest nanoparticle accumulation, followed by the spleen, although with fifteen times less uptake than the liver. The same behavior was observed in the histological results of this research since nanoparticle clusters were only observed in the liver and spleen of treated mice but without induction of tissue toxicity (Figure 5). A detailed analysis of liver histology showed that in both treated and control groups, hepatocytes were displayed in plates, some of which have minor degenerative signs. The typical hepatic blood vessel containing erythrocytes and small leukocytes was appreciated.

The liver of mice treated with [^177^Lu]Lu−iFAP/iPSMA nanoparticles exhibited no histopathological abnormalities. No granulomas or hemorrhages were observed. There was a slight lymphocytic infiltration (mainly Kupffer cells). The nanoparticle clusters in treated mice were not found in endothelial cells of the hepatic parenchyma but were observed near blood vessels and in Kupffer cells (Figure 5). In the spleen micrographs, nanoparticle clusters were neither observed in the splenic red pulp (erythrocytes and lymphocytes) nor in endothelial cells, but were found in macrophages (Figure 5).

**Figure 6:**
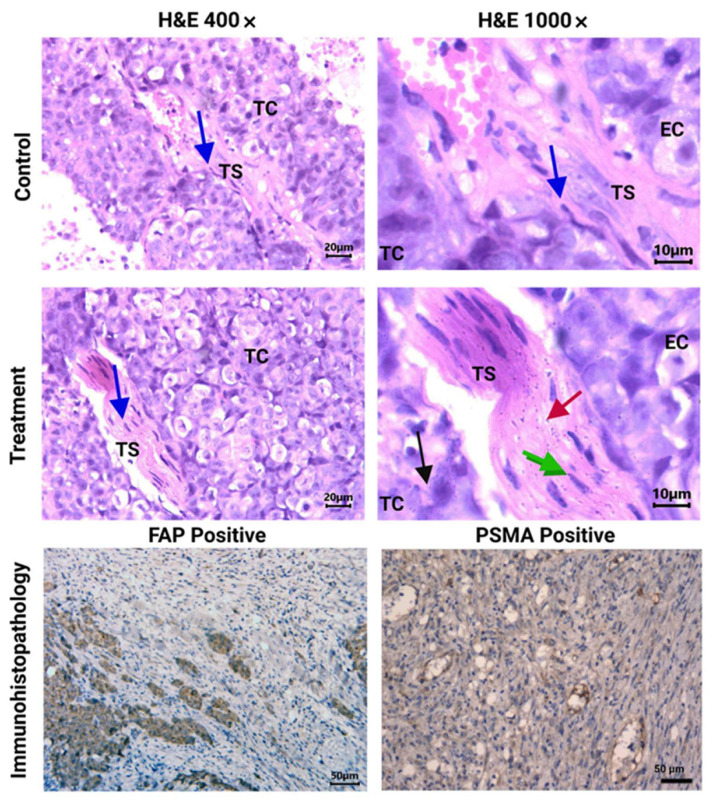
Histopathological analysis (H&E staining). Light micrographs of representative sections at 400× and 1000× magnification of HCT116 tumors from [^177^Lu]Lu−iFAP/iPSMA-treated and untreated (control) mice. Cancer-associated fibroblasts (CAFs) can be observed in the tumor stroma (TS) (blue arrow). Note nanoparticle clusters of [^177^Lu]Lu−iFAP/iPSMA in TS (red arrow), in CAFs (green arrow), and in tumor cells (TC) (black arrow), but not found in endothelial cells (EC). PSMA and FAP expression in HCT116 tumors (dark brown color) (200× magnification of) is also shown (immunohistopathology results).

In contrast, nanoparticle clusters of [^177^Lu]Lu−iFAP/iPSMA were abundantly observed in tumor stroma (including tumor neovasculature), cancer-associated fibroblasts, and tumor cells, but not found in endothelial cells (Figure 6). Moreover, [^177^Lu]Lu−iFAP/iPSMA induced tumor necrosis. The results obtained can be correlated with the three main mechanisms of selective tumor uptake associated with nanoparticles: (1) high uptake in the tumor stroma due to the enhanced permeability and retention (EPR) effect (a passive mechanism); (2) expression of FAP in fibroblasts; and (3) PSMA expression in cancer cells and tumor neovasculature, which involves active mechanisms mediated by receptors present in the tumor stroma that recognize iFAP and iPSMA ligands bound to the surface of the nanoparticles. These results were confirmed by the immunohistochemistry evaluation, which revealed FAP+ and PSMA+ expression on colorectal cancer cells of xenograft tumors (Figure 6).

The creatinine, ALT, AST, LDH, and CK blood serum values of 0.19 ± 0.04 mg/dL, 68 ± 7 IU/L, 130 ± 12 IU/L, 291 ± 32 IU/L, and 120 ± 13 IU/L, respectively, obtained after treatment with [^177^Lu]Lu−iFAP/iPSMA nanoparticles in mice (without significant difference among the blood values of control mice: *p* > 0.05), also confirmed the absence of healthy tissue injury.

**Figure 7:**
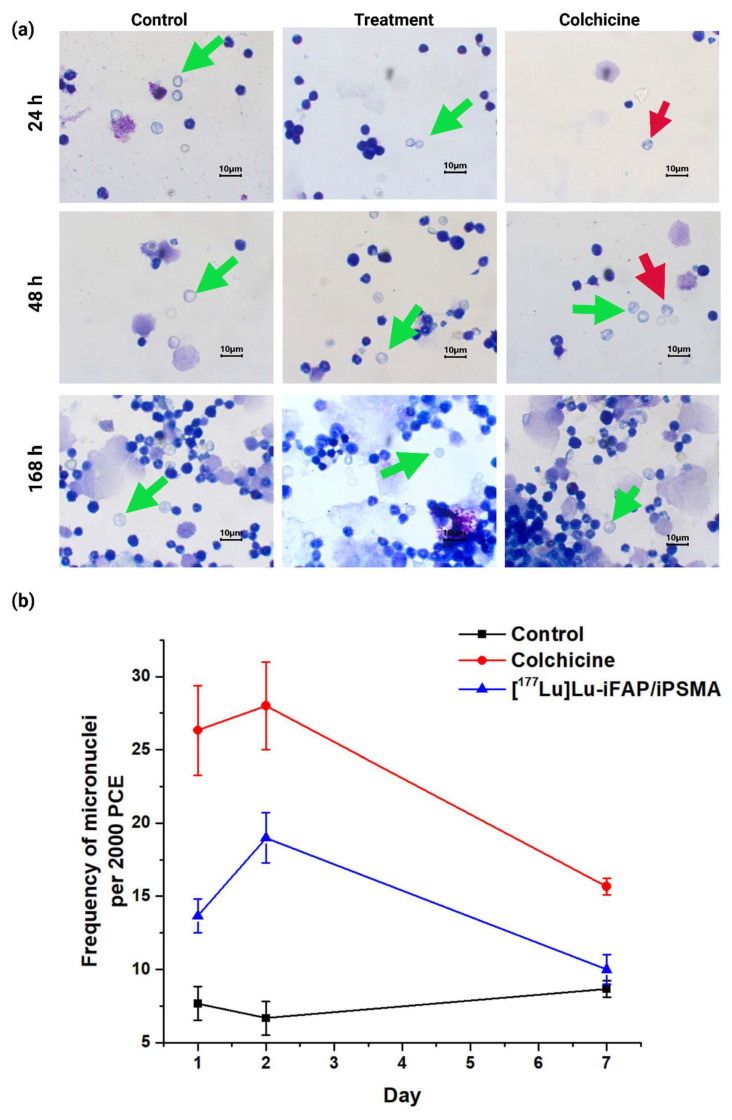
(**a**) Light micrographs (Giemsa-Wright staining at 1000× magnification of) of bone marrow cells from healthy mice at 24, 48, and 168 h after i.v. administration of [^177^Lu]Lu−iFAP/iPSMA nanoparticles (treatment), colchicine (1 mg/kg) (positive control), and untreated mice (negative control). Green arrows show polychromatic immature erythrocytes, and red arrows indicate polychromatic erythrocytes (PCE) with micronuclei. (**b**) Micronucleus count values in immature bone marrow erythrocytes at 1, 2, and 7 d. Data are presented as mean ± standard deviation of three independent experiments. Non-significant difference (*p* > 0.05) between control and treatment at 7 d.

The micronuclei assay helped to evaluate the possibility of any genetic instability induced by the [^177^Lu]Lu−iFAP/iPSMA nanoparticles. Compared to the negative control at 1 and 2 d post-treatment, [^177^Lu]Lu−iFAP/iPSMA apparently produced a genotoxic effect (*p* < 0.05) (Figure 7). However, after 7 d, no significant difference was observed between the negative control and the treatment with lutetium radionanoparticles (*p* > 0.05). Therefore, the observed toxicity can be attributed to a well-known and transient toxic effect of radiation on the bone marrow [38,40], but not to the nanoparticles themselves. That is, if the activation of DNA repair mechanisms was not sufficiently effective due to severe nanoparticle damage, the expected behavior would be, at least, like that of the positive control (colchicine), which did not occur. Furthermore, it is essential to consider that the dose injected into mice was 60 times higher (~0.850 mg of nanoparticles (740 MBq)/kg) than recommended in patients (~0.014 mg of nanoparticles (12 MBq)/kg) after a dosimetric evaluation [27].

## 4. Discussion

One of the most important current challenges in producing therapeutic nanoparticles for application in patients is the development of efficient processes that meet the requirements established by the regulatory authorities in terms of GMP. In this research, [^177^Lu]Lu−iFAP/iPSMA nanoparticles were reproducibly prepared, under GMP conditions, as sterile colloidal solutions free of bacterial endotoxins and with high radiochemical and radionuclidic purity, which adequately complied with the quality characteristics of a pharmaceutical grade formulation suitable for clinical use (Table 1) [32].

Another challenge to overcome is the toxicity associated with nanoparticles, which depends on their chemical composition, size and shape, surface coating, surface area, and in vivo stability [28]. In general, the mechanism by which nanoparticles cause toxicity lies in their ability to initiate the formation of genotoxic and cytotoxic reactive species associated with increased oxidative stress and inflammation (immune-mediated effects) [29].

The toxic effects of metallic nanoparticles in tissues have been among the most studied. Considering that the primary uptake is by the reticuloendothelial system led by the liver and spleen, it has been reported that nanoparticles induce structural and dysfunctional hepatic changes associated with increased levels of proinflammatory cytokines, such as IL-1β and IL-6, as well as an augmentation in alkaline phosphatase, AST, and ALT [41,42,43,44]. In addition, autophagy, pyroptosis, apoptosis, necrosis, and lipid peroxidation (induced by reactive oxygen species) are pathways of hepatocyte injury and death attributed to the high toxicity of nanoparticles [29,42,44].

However, specific differences make [^177^Lu]Lu−iFAP/iPSMA nanoparticles a safe option for potential use in the treatment of patients. First, the functionalization on their surface with biomolecules (ligands based on biopeptides) could result in the non-recognition of the nanoparticles as foreign elements by the immune system. Secondly, due to the steric effect of the ligands attached to the nanoparticles, chemical interactions that could induce the formation of reactive oxygen species were minimized. As shown in Figure 4, healthy tissues showed no signs of inflammation (lymphocytic infiltration), granuloma formation, hemorrhage, necrosis, or fibrotic processes. In fact, the only mechanism of cellular uptake of the radionanosystem in the spleen and liver was found through the macrophages (Figure 5). However, macrophages are highly radio-resistant to the extent that they can repair DNA double-strand breaks [38,39]. Increased Bcl-xL expression and NF-kappa B transcription activation have been reported as the mechanism of macrophages’ pro-survival [39]. In addition, irradiated macrophages are viable, metabolically active, present increased phagocytosis, and have a reduced anti-inflammatory profile (decreased expression of CD163, IL-10, VCAN, and MRC1) [39]. Although the phagocytosis of metallic nanoparticles by macrophages induces a proinflammatory mechanism [42] in the immune system, it was not observed in healthy tissues after [^177^Lu]Lu−iFAP/iPSMA treatment (Figure 4 and Figure 5), which can also be attributed to its biocompatibility mediated by the ligands that cover the nanoparticle surface.

The genotoxicity associated with the bone marrow irradiation was transient since a recovery in mice treated with [^177^Lu]Lu−iFAP/iPSMA was observed after 7 d [38,40]. Therefore, genotoxicity was not observed as a consequence of an excess of free radicals induced from nanoparticles due to oxidative stress with a potential mutagenic effect [28,29], which confirmed that intravenous systemic administration is safe.

At this point, it is essential to emphasize that the first mechanism of lutetium nanoparticle uptake by tumors is the EPR effect, which takes advantage of the size of the endothelial intercellular space (from 400 nm to 800 nm) of tumors, in contrast to 2 nm between endothelial cells of healthy tissues [45]. Furthermore, within the tumor, the [^177^Lu]Lu−iFAP/iPSMA nanosystem is additionally retained by a mechanism mediated by FAP and PSMA proteins (Figure 6) [27]. In such a way, the non-malignant cells of normal healthy organs have minimal opportunity to be in contact with the administered nanoparticles, reducing the potential nanosystem toxicity. Recently, Luna-Gutierrez et al. [27] showed, through nuclear molecular imaging, that after intravenous [^177^Lu]Lu-iPSMA nanoparticle administration to a patient with multiple colorectal liver metastases, a highly selective uptake by tumor lesions occurred with minimal visualization of the liver parenchyma. That is, healthy hepatic parenchyma cells did not trap lutetium nanoparticles. Therefore, the [^177^Lu]Lu−iFAP/iPSMA nanosystem could be a safe option for future clinical protocols in treating hepatic tumors, particularly colorectal liver metastases. Furthermore, futures clinical studies could also be carried out in combination with novel anticancer immunotherapies focused on inhibiting metastasis, therapeutic resistance, and cancer progression by reducing immunosuppression and remodeling the tumor microenvironment [2].

## 5. Conclusions

The data obtained in this research provide new evidence on the selective toxicity to malignant tumors and the absence of histological changes in healthy tissues after intravenous administration of [^177^Lu]Lu−iFAP/iPSMA in mammalian hosts. The easy preparation under GMP conditions and the toxicity features provide the added value for [^177^Lu]Lu−iFAP/iPSMA clinical translation in treating colorectal liver metastases.

## Figures and Tables

**Table 1:** Quality control analytical results of [^177^Lu]Lu−iFAP/iPSMA nanoparticles produced under GMP conditions (results of three batches).

Test	Specification	Method	Results
Activity per vial	3.40 ± 0.20 GBq	Ionization chamber	3.45 ± 0.10 GBq
Radioactive concentration	170 ± 8 MBq/mL	Ionization chamber	170 ± 5 MBq/mL
Specific activity	≥0.8 GBq/mg Lu	TXRF/ Ionization chamber	0.86 ± 0.4 GBq/mg
Identity of ^177^Lu	Main emission peaks E_ϒ_ = 113 keV; 208 keV	Gamma spectrometry	Complies
Radionuclidic purity: ^177m^Lu Other impurities	≤0.02% ≤0.01%	Gamma spectrometry	0.009 ± 0.001% ≤0.004%
Chemical purity	Co, Ni ≤ 0.1 µg/GBq Cu, Pb ≤ 0.5 µg/GBq Fe, Zn ≤ 1.0 µg/GBq	ICP-MS/ Ionization chamber	Ni, Cu < 0.020 µg/GBq Co < 0.001 µg/GBq Pb < 0.068 µg/GBq Zn < 0.074 µg/GBq Fe < 0.152 µg/GBq
Lutetium content	80–120 mg/L	TXRF	102 ± 21 mg/L
pH value	6 ± 1	pH indicator strips	6
Radiochemical purity	>98%	Ultracentrifugation	>99%
Sterility test	Sterile	Sterility test [32]	Sterile
Bacterial endotoxin	<20EU/mL	Bacterial endotoxin test [32]	Complies

## Data Availability

Not applicable.
